# Purification and Identification of the Nematicidal Activity of S1 Family Trypsin-Like Serine Protease (PRA1) from *Trichoderma longibrachiatum* T6 Through Prokaryotic Expression and Biological Function Assays

**DOI:** 10.3390/genes15111437

**Published:** 2024-11-06

**Authors:** Nan Ma, Hang Lv, Solomon Boamah, Shuwu Zhang, Bingliang Xu

**Affiliations:** 1 College of Plant Protection, Gansu Agricultural University, Lanzhou 730070, China; manan1125@163.com (N.M.); lvhanng@126.com (H.L.); boamahsolomon15@gmail.com (S.B.); xubl@gsau.edu.cn (B.X.); 2 Biocontrol Engineering Laboratory of Crop Diseases and Pests of Gansu Province, Lanzhou 730070, China; 3 Gansu Provincial Key Laboratory of Arid Land Crop Science, Gansu Agricultural University, Lanzhou 730070, China

**Keywords:** *Trichoderma longibrachiatum* T6, *Heterodera avenae*, serine protease, function identification, biological control

## Abstract

Background/Objectives: *Heterodera avenae* is a highly significant plant-parasitic nematode, causing severe economic losses to global crop production each year. *Trichoderma* species have been found to parasitize nematodes and control them by producing enzymes that degrade eggshells. The *T. longibrachiatum* T6 (T6) strain has been demonstrated the parasitic and lethal effects on *H. avenae* cysts and eggs, associated with the increased serine protease activity and trypsin-like serine protease gene (*PRA1*) expression. Methods: Our present study aimed to purify the recombinant PRA1 protease through a prokaryotic expression system and identify its nematicidal activity. Results: The recombinant PRA1 protease was identified as S1 family trypsin-like serine protease, with a molecular weight of 43.16 kDa. The purified soluble protease exhibited the optimal activity at 35 °C and pH 8.0, and also demonstrating higher hydrolytic ability toward casein and skimmed milk. Meanwhile, the Ca^2+^ and Mg^2+^ enhanced its activity, while the inhibitor PMSF significantly reduced it. The contents of *H. avenae* eggs leaked out after treatment with the recombinant PRA1 protease, with egg hatching inhibition and relative hatching inhibition rates at 70.60% and 66.58%, respectively. In contrast, there was no sign of content dissolution, and embryos developed normally in the control group. Conclusions: Our present study revealed that the PRA1 protease of T6 strain has a lethal effect on *H. avenae* eggs, which providing a theoretical basis for developing biocontrol agents to control nematodes.

## 1. Introduction

Cereal cyst nematodes (CCNs) are among the most significant plant-parasitic nematodes that affecting the cereal crops including wheat and barley [1]. In wheat, yield losses due to CCNs can vary depending on environmental conditions, with severe infestations reducing yields and even causing total crop failure [2]. However, it is very difficult to control CCNs due to their eggs are protected by cysts. This protection shields the eggs from parasites and desiccation, allowing them to remain dormant for years, which complicates the development of effective control strategies [3]. Chemical control techniques, in general, mostly rely on synthetic chemicals to reduce the CCN population densities [4]. Controlling nematodes with chemicals becomes more challenging, as nematicides must be non-toxic to plants and ideally systemic to be effective, and even sometimes the extensive use of chemicals to control plant-parasitic nematodes is detrimental to human health and the environment [5]. Therefore, chemical control of CCNs has become a significant challenge worldwide, calling for alternative, sustainable, and eco-friendly biocontrol strategies urgently.

*Trichoderma* species are effective biocontrol fungi for managing nematode diseases through direct antagonism and mycoparasitism, whereby they parasitize and degrade nematode cells or eggs by enzymes like chitinases and proteases [6,7]. Fermentation broths from *Trichoderma citrinoviride*, *Trichoderma harzianum*, *Trichoderma atroviride*, and *Trichoderma koningiopsis* showed nematicidal activity on *Meloidogyne incognita* juveniles, with mortality rates of up to 85% [8,9,10]. *Trichoderma virens* also exhibits 86.2% mortality against *H. avenae* by disrupting cysts [11], and also *Trichoderma koningii*, *Trichoderma viride*, and *T. harzianum* strains reduce *Meloidogyne* spp. egg hatching and kill juveniles [12].

Mycoparasitism by *Trichoderma* species is a key mechanism for controlling nematodes, where the fungi secrete various hydrolytic enzymes such as chitinases and proteases that play the dominant role in degrading nematode eggshells and cuticles [13]. Research by Szabo et al. [7] demonstrated that *Caenorhabditis elegans* eggs can significantly induce the upregulation of *T. harzianum* chitinase genes *chi18-5* and *chi18-12*, further enhancing the production of chitinase. Sahebani et al. [14] also found that crude chitinase extract from *T. harzianum* could inhibit the hatching of *M. javanica* eggs, degrade their eggshells, and prevent them from hatching into second-stage juveniles (J2s). Additionally, the protease PRA1 from *T. harzianum* CECT 2413 that can degrade fungal cell wall proteins and inhibit *M. incognita* eggs hatching [15], and the use of a proteinase Prb1-transformed line had the ability to penetrate egg masses and colonize the eggs compared with the wild-type strain [16]. Similarly, the *prb1* gene from *T. harzianum* and its overexpression mutant showed higher nematicidal activity against *M. incognita* [17]; a novel serine protease, SprT, from *T. pseudokoningii* strain SMF2, was also identified, and found that can significantly reduce the *M. incognita* egg hatching, and exhibiting toxicity against J2s [18].

In our previous study, *T. longibrachiatum* T6 (T6) presented the parasitic and lethal effects on *H. avenae* cysts, eggs, and juveniles. Microscopic examination also demonstrated that the dissolved and ruptured cysts, eggs, and juveniles of *H. avenae*, which may attributed to the chitinase and protease secretion of T6 strain [19]. In the process of interaction between *H. avenae* and T6, there was a significant upregulation of the trypsin-like serine protease gene *PRA1* expression of T6 strain. Though T6 has shown potential as a nematicide, little is known about its serine proteases. Thus, our present study aims to express, purify, and analyze the enzymatic properties of recombinant PRA1 protease and identify its nematicidal activity.

## 2. Materials and Methods

### 2.1. Experimental Design

This study aimed to purify and identify the nematicidal activity of a trypsin-like serine protease (PRA1) from *T. longibrachiatum* T6 (T6) through prokaryotic expression and biological function assays. The experimental design included excluding the *PRA1* gene signal peptide, and inserting into the pET-32a vector, which was then expressed in *Escherichia coli* BL21 (DE3) cells and induced with IPTG for recombinant protease production. The expressed PRA1 was subsequently purified using His-tag affinity chromatography. Following purification, enzymatic characterization was conducted to assess the optimal temperature, pH, metal ion effects, and substrate specificity of the recombinant PRA1. Finally, biological function assays were performed to evaluate the nematicidal activity of PRA1 on *H. avenae* eggs, specifically examining its efficacy in degrading nematode eggs and preventing its hatching.

### 2.2. Samples Preparation

The T6 strain (CGMCC No. 13183) was provided by the Laboratory of Plant Virology and Molecular Biology, Gansu Agricultural University. Fresh cysts of *H. avenae* were sampled from the rhizosphere soil of wheat severely affected by cereal cyst nematodes in Suzhou, Anhui Province. The cysts were isolated and egg suspensions were prepared using the method described by Zhang et al. [19].

### 2.3. Prokaryotic Expression of the PRA1 Gene and Protein Induction

Based on the *PRA1* gene (GenBank ID: PQ373860) sequence of T6 and the pET-32a vector (Miaoling Biology, Wuhan, China) sequence, primers YF and YR (Table 1) were designed. The primer YF was added with *BamH* I restriction site, and the primer YR was added with *Xho* I restriction site. Using cDNA from T6 as template, and YF and YR as primers, the *PRA1* gene fragment without signal peptide was amplified using high-fidelity polymerase (Takara Biomedical Technology (Beijing) Co., Ltd., Beijing, China). The gene fragment and vector were digested with *BamH* I and *Xho* I (Takara Biomedical Technology (Beijing) Co., Ltd., Beijing, China), and ligated with T4 DNA ligase (Takara Biomedical Technology (Beijing) Co., Ltd., Beijing, China) and transformed into *E. coli* DH5α cells (Takara Biomedical Technology (Beijing) Co., Ltd., Beijing, China). The transformed cells were plated on LB solid medium that containing ampicillin at a final concentration of 100 μg/mL and then incubated overnight at 37 °C. Positive transformants were selected, and single colonies were picked for colony PCR verification and double-enzyme digestion analysis. The colony PCR verification used T7 and T7ter as primers. The positive recombinant plasmid was named pET-32a-PRA1 after successful verification and transformed into *E. coli* BL21(DE3) competent cells (Biomed Technology, Beijing, China). Positive colonies were inoculated into LB liquid medium containing ampicillin at a final concentration of 100 μg/mL and cultivated at 37 °C with shaking at 200 rpm until the culture’s OD600 values reached 0.4 to 0.6. The culture was induced at 28 °C with shaking at 200 rpm for 4 h after IPTG was added to a final concentration of 0.25 mmol/L. The *E. coli* BL21(DE3) cells containing the empty pET-32a vector induced with IPTG for 4 h and the its cells containing the pET-32a-PRA1 vector uninduced were used as controls, respectively.

### 2.4. Protein Expression Verification and Molecular Weight Determination by SDS-PAGE Analysis

SDS-PAGE (12%) (Solarbio, Beijing, China) was used to confirm the successful expression of recombinant PRA1. Samples from both induced and uninduced cultures were prepared. Briefly, cultures without IPTG induction were used as controls, and cultures with IPTG induction containing the pET-32a-PRA1 vector were used as the treatment. Following induction, the cultures were centrifuged at 12,000 rpm and 4 °C for 5 min. The remaining liquid was then discarded, and the cell pellet was extracted. For each milliliter of bacterial pellet, 100 μL of lysis buffer (Beyotime Biotechnology, Shanghai, China) was added to fully lyse the pellet, and then it was centrifuged for 5 min to separate the supernatant and pellet. Protein loading buffer was added, mixed well, and then heated at 100 °C in a water bath for 10 min to denature the protein. After cooling, it was centrifuged at 12,000 rpm for 5 min, and the supernatant was loaded onto the gel for electrophoresis. The presence of the PRA1 protease was confirmed based on its molecular weight in comparison to the protein marker. The recombinant PRA1 protease was purified using His-tag affinity chromatography under denaturing conditions using a purification kit (denatured type) (P2229S, Beyotime Biotechnology, Shanghai, China). The purified protease was collected for further enzymatic and biological function analyses.

### 2.5. Enzymatic Characterization

#### 2.5.1. Optimal Temperature and Thermal Stability Determination

The Folin phenol method [20] was utilized to quantify the protease activity, with casein as the substrate. The amount of enzyme required to catalyze the hydrolysis of 1 μmol of tyrosine per minute per milligram of protein was determined as one unit of enzyme activity. The water bath’s reaction temperature was adjusted to 15, 20, 25, 30, 35, 40, 45, 50, 55, and 60 °C, and the enzyme activity was measured at each setting. The optimal protease temperature was determined by calculating the relative enzyme activity at each temperature, with casein serving as the substrate and the maximum enzyme activity set at 100%. The protease solution was incubated in a water bath at 15, 20, 25, 30, 35, 40, 45, 50, 55, and 60 °C for 30 min in order to evaluate its thermal stability. The remaining enzyme activity was then evaluated at the optimal temperature. The protease’s heat stability was assessed by calculating the relative enzyme activity, with the maximum enzyme activity being set at 100%. Three duplicates of each treatment were used.

#### 2.5.2. Optimal pH and pH Stability Determination

The pH of the reaction was set to 3, 4, 5, 6, 7, 8, 9, 10, 11, and 12 at the ideal temperature. Calculations were made to determine the enzyme activity at each pH, with 100% being the maximum enzyme activity. To find the optimal pH of the protease, the relative enzyme activity at each pH was estimated. In order to evaluate pH stability, the protease solution was incubated for 30 min at the optimal temperature in buffers with the pH value ranging from 3 to 12. Afterwards, ideal conditions were met for measuring the residual enzyme activity. The protease’s pH stability was assessed by calculating the relative enzyme activity, with the maximum enzyme activity set at 100%. Each treatment was performed in triplicate.

#### 2.5.3. Effects of Metal Ions and Inhibitors on Recombinant Protease Activity

At a final concentration of 5 mmol/L, metal ions (Fe^2+^, Fe^3+^, Cu^2+^, Mn^2+^, Ca^2+^, Mg^2+^, Na^+^, K^+^, Li^+^, Zn^2+^) were added to the reaction system. In the meantime, the inhibitor EDTA with a final concentration of 5 mmol/L and the inhibitor PMSF with a final concentration of 1 mmol/L were added to the reaction system respectively. The pH of 8.0 and temperature of 35 °C were the reaction conditions used to test the enzyme activity. To assess the impact of metal ions and inhibitors on the recombinant protease, the relative enzyme activity was computed, with the initial enzyme activity set at 100% in the absence of these substances. Each procedure was carried in triplicate.

#### 2.5.4. Effects of Different Substrates on Recombinant Protease Activity

The enzyme activity was measured using 1% casein, skimmed milk, bovine serum albumin (BSA), gelatin, and nematode eggshells (*H. avenae*) as substrates under reaction conditions of 35 °C and pH 8.0. The enzyme activity with casein as the substrate was set as 100%, and the relative enzyme activity was calculated to assess the hydrolytic activity of the recombinant protease on various protein substrates. Each treatment was performed in triplicate.

### 2.6. Biological Function Assays

The nematicidal activity of recombinant PRA1 protease was tested on *H. avenae* eggs. Sterile 96-well plates were prepared with 10 μL of egg suspensions (3 ± 1 eggs per well) and treated with 190 μL of purified PRA1 following the method described by Shen et al. [21]. The *E. coli* BL21(DE3) cells containing the empty pET-32a vector induced with IPTG for 4 h and sterile water were used as positive and negative controls, respectively. The cell culture plates were incubated in a constant-temperature incubator at 16 °C for 10 days. Microscopic observation and photography were performed every 2 days to observe the morphological characteristics of the eggs after treatment with purified PRA1. The number of hatched eggs was recorded at 10 days after treatment with purified PRA1. Each treatment and control were replicated 36 times. The percentages of egg hatching inhibition and the relative egg hatching inhibition were determined using the Equations (1) and (2):

Egg hatching rate = (number of hatched eggs)/(total number of eggs) × 100%(1)

Relative egg hatching inhibition rate = (control hatching rate − treatment hatching rate)/(control hatching rate) × 100%(2)

### 2.7. Statistical Analysis

In the present study, the data was expressed by the means ± standard errors (SE) of independent experiments. The software of the SPSS version 26.0 (IBM Corp., Armonk, NY, USA) was applied to conduct the statistical analysis. One-way data analysis of variance (ANOVA) was used to analyze statistical differences based on the Duncan’s multiple range test at *p* < 0.05.

## 3. Results

### 3.1. Construction of Prokaryotic Expression Vector of the PRA1 Gene

**Figure 1:**
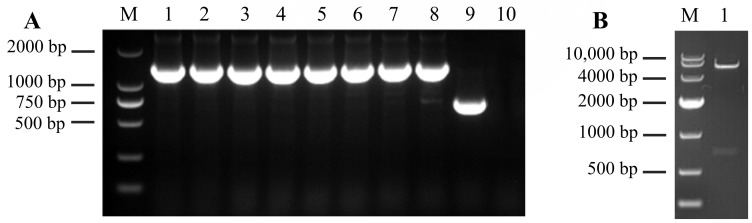
Construction of the expression vector with 1.2% agarose gel electrophoresis detection: (**A**) The *E. coli* DH5α detection using PCR, where M represents the 2000 DNA Marker, lanes 1–8 are pET-32a-PRA1 recombinant plasmid that has been transformed to *E. coli* DH5α, lane 9 is the pET-32a empty plasmid amplification (positive control), and lane 10 is the sterile water instead of template amplification (negative control). (**B**) Dual-enzyme digestion verification of the recombinant plasmid pET-32a-PRA1, where M represents the 10,000 DNA Marker, and lane 1 represent the double-enzyme digestion products of the recombinant plasmid pET-32a-PRA1.

The *PRA1* gene was inserted into the pET-32a plasmid, and the recombinant construct was verified in the present study. The results showed that the band size of more than 1000 bp in each of 8 replication colonies that containing the recombinant plasmid, close to the expected size of 1392 bp (Figure 1A, lanes 1–8). In contrast, the colony with the empty pET-32a vector produced a 750 bp band in the positive control (Figure 1A, lane 9). However, no band appeared in the negative control (Figure 1A, lane 10). Further validation was performed by extracting the recombinant plasmid and performing double digestion. Two bands of 5860 bp and 720 bp were observed (Figure 1B, lane 1), which matching with the predicted sizes. The successfully constructed plasmid was named pET-32a-PRA1.

### 3.2. Expression and Purification of Recombinant PRA1 Protease

**Figure 2:**
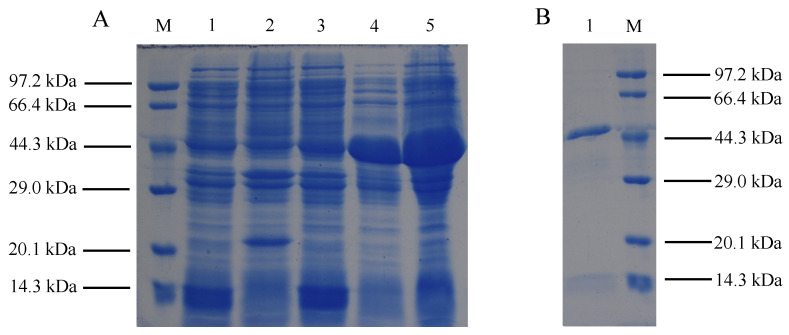
Determination of recombinant PRA1 protease expression by SDS-PAGE: (**A**) Induction of recombinant PRA1 protease in *E. coli* BL21 (DE3) using IPTG, where M represents the protein marker; the first lane is a non-induced pET-32a empty vector at 28 °C for 4 h; the second lane represents a positive control, which is an *E. coli* BL21 (DE3) solution of pET-32a empty vector that has been transformed into competent cells and induced at 28 °C for 4 h; the third lane displays an *E. coli* BL21 (DE3) solution of recombinant protease that has not been induced; the fourth lane represents a supernatant of recombinant protease that has been induced at 28 °C for 4 h; and the fifth lane represents precipitation of recombinant protease that has been induced at 28 °C for 4 h. (**B**) Analysis of the isolated recombinant protease using SDS-PAGE, where lane 1 is the purified recombinant protease and M is the protein marker.

The successfully constructed vector was extracted from *E. coli* DH5α and transformed into *E. coli* BL21 (DE3) competent cells that were optimized for high-level protein expression. The predicted molecular weight of the recombinant PRA1 protease, with 6× His tags at both ends and no signal peptide, was 43.16 kDa. SDS-PAGE analysis revealed that no protein expression around 43.16 kDa in cultures containing the empty vector or in uninduced recombinant plasmid cultures (Figure 2A, lanes 1–3).In contrast, the protein bands around 43.16 kDa appeared in both the supernatant and the pellet after IPTG induction at 28 °C for 4 h, which indicating that the successful expression of the *PRA1* gene (Figure 2A, lanes 4–5). Following purification using a His-tag kit, a single band at 43.16 kDa was observed (Figure 2B, lane 1), indicating effective isolation of the recombinant PRA1 protease. This result validated the successful expression and purification of PRA1 protease.

### 3.3. Determination of the Enzymatic Properties of Recombinant PRA1 Protease

**Figure 3:**
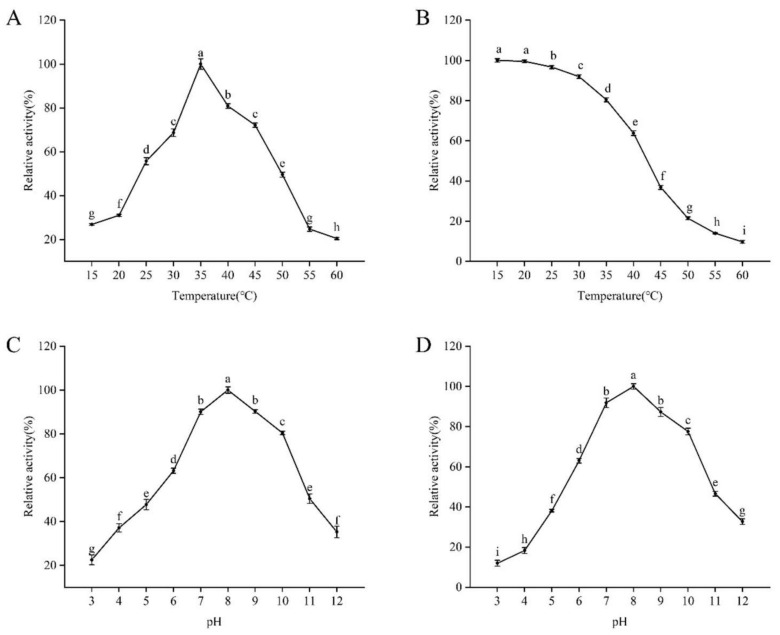
Effects of temperature and pH on the recombinant PRA1 protease’s enzyme activity: (**A**) Recombinant PRA1 protease at its optimal temperature. (**B**) The recombinant PRA1 protease’s thermal stability. (**C**) The recombinant PRA1 protease at its optimal pH. (**D**) The pH stability of the recombinant PRA1 protease. The means ± standard errors are displayed for the data, and columns labeled with various letters denote significant differences at *p* < 0.05.

The optimal temperature and thermal stability assays revealed that the enzyme had the highest activity at 35 °C. Enzyme activity increased from 15 °C to 35 °C, peaking at 35 °C, and then decreased significantly. Notably, it retained over 68.71% of its activity between 30 °C and 45 °C, demonstrating robust performance across various temperatures, which is advantageous for practical applications (Figure 3A). Thermal stability assessments showed that the enzyme was most stable at 15 °C, with stability decreasing at higher temperatures. However, it still retained over 63.83% of its activity from 15 °C to 40 °C, indicating functionality under different conditions (Figure 3B).

The optimal and stability pH for enzyme activity was found to be pH 8.0 (Figure 3C,D), with activity increasing from pH 3.0 to 8.0 before declining. The enzyme maintained over 63.17% of its activity across a pH range of 6.0 to 10.0, suggesting stability in slightly alkaline conditions. Its maximum stability was also observed at pH 8.0.

### 3.4. Effects of Metal Ions and Inhibitors on Recombinant Protease Activity

The effects of different metal ions and inhibitors on enzyme activity were measured under the optimal reaction conditions (35 °C and pH 8.0) (Table 2). Compared with the control, Fe^2+^, Fe^3+^, Cu^2+^, and Mn^2+^ all exhibited inhibitory effects on enzyme activity, with relative activities of 41.80%, 39.70%, 62.56%, and 69.17%, respectively. In contrast, Ca^2+^ and Mg^2+^ promoted the enzyme activity significantly, with relative activities of 133.83% and 123.16%, respectively. Na^+^, K^+^, Li^+^, and Zn^2+^ showed no significant effects on enzyme activity, suggesting they do not influence its function under the tested conditions.

However, the serine-protease-specific inhibitor PMSF exhibited strong inhibitory effects, reducing the enzyme activity by more than 70% at a concentration of 1 mmol/L, confirming the enzyme’s classification as a serine protease and indicating the presence of a critical serine residue in its active site.

### 3.5. Effects of Different Substrates on Recombinant Protease Activity

The hydrolytic activity of the recombinant protease on different protein substrates was measured under the optimal reaction conditions (35 °C and pH 8.0) (Table 3). The recombinant PRA1 protease displayed the highest hydrolytic activity toward casein, followed by skimmed milk. Using casein as the substrate, the protease activity was set to 100%, and the relative activity was 78.94% with skimmed milk as the substrate. It also demonstrated a lower hydrolytic activity on bovine serum albumin (BSA), gelatin, and nematode eggshells.

### 3.6. Microscopic Observation of Eggs Treated with Recombinant Protease

**Figure 4:**
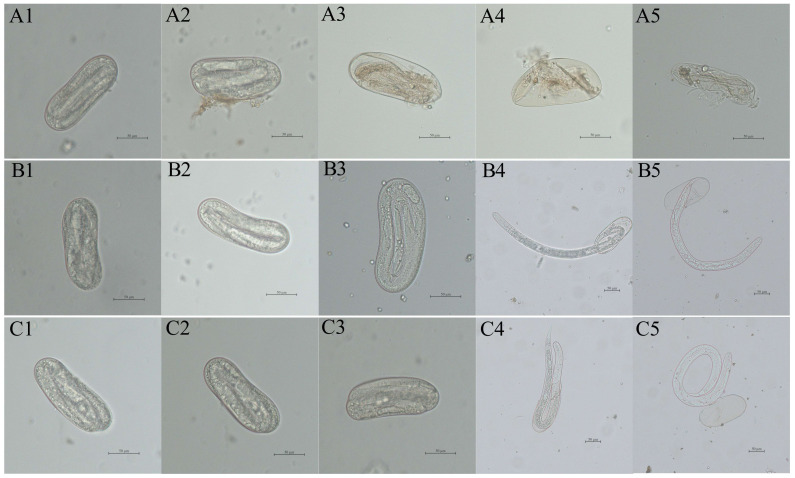
Morphological analysis of *H. avenae* eggs subjected to recombinant PRA1 protease treatment. Eggs after treatment with recombinant PRA1 protease are represented in images (**A1**–**A5**), taken at 2, 4, 6, 8, and 10 days under 400× magnification, respectively. Samples (**B1**–**B5**) served as positive controls, representing eggs at 2, 4, 6, 8, and 10 days post-treatment with the *E. coli* BL21 (DE3) solution containing the pET-32a empty vector, which was transformed into competent *E. coli* BL21 (DE3) cells, respectively. Observations for (**B1**–**B3**) were conducted at a magnification of 400×, while (**B4**,**B5**) were observed at 200× magnification. (**C1**–**C5**) served as negative controls, representing eggs at 2, 4, 6, 8, and 10 days post-treatment with sterile water, respectively. Observations for (**C1**–**C3**) were conducted at 400× magnification, while (**C4**,**C5**) were examined at 200× magnification.

Microscopic observations of the eggs of *H. avenae* treated with the purified recombinant PRA1 protease (with enzymatic activity of 2.26 U/mL and a concentration of 1.14 mg/mL) showed that the eggshells became thinner, partially dissolved, and a small amount of egg content leaked out (Figure 4A2). By days 8 and 10 post-treatment, significant egg content leakage caused the eggshells to shrink into empty shells, preventing normal hatching (Figure 4A4,A5). In contrast, the embryos developed normally at days 2 to 6 in control groups (Figure 4B1–B3,C1–C3). From the 8th day post-treatment, J2s began to hatch from the eggs with intact body walls (Figure 4B4–B5,C4–C5).

### 3.7. Inhibitory Effect of Recombinant Protein on Egg Hatching

On the 10th day after treatment with purified recombinant PRA1 protease, the hatching inhibition rate of the eggs reached 70.60%. In contrast, the hatching inhibition rates of the eggs treated with the *E. coli* BL21 (DE3) solution containing the pET-32a empty vector (positive control) and sterile water (negative control) were only 10.88% and 12.04%, respectively, which were significantly lower than that of the PRA1 protease treatment. Additionally, on the 10th day after treating the eggs with recombinant PRA1 protease, the relative hatching inhibition rate reached 66.58% (Table 4).

## 4. Discussion

*Trichoderma* species are well known for their biocontrol capabilities, exhibiting antagonism against a wide range of plant pathogens, including fungi and nematodes [22,23]. *Trichoderma* parasitizes plant-parasitic nematodes by secreting a range of extracellular hydrolytic enzymes that degrade eggshells and cuticles, which was considered to be the key factors in the biological control of nematodes [24].

Studies showed that the serine protease genes *TghSS42* from *T. ghanense* [25] and *ThSS45* from *T. harzianum* [26] were successfully expressed in *E. coli* with the molecular weight of 68.5 kDa and 69 kDa, respectively. In addition, the serine protease SprT from *T. pseudokoningii* SMF2 have been reported with molecular weights of 31 kDa, which had nematicidal activity [18]. In this study, the recombinant PRA1 protease from *T. longibrachiatum* T6 (T6), which has 6× His tags at both the N- and C-termini, exhibited a molecular weight of 43.16 kDa. 

In the current study, the optimal temperature of the recombinant PRA1 protease was 35 °C, and the optimal pH was 8. It was relatively stable in the temperature range of 30–45 °C, with a relative activity greater than 60%. In comparison with previous study, the *T. harzianum* PRA1 protease exhibited only 16% activity at 45 °C [15]; The SprT protease from *T. pseudokoningii* SMF2 demonstrated good thermal stability and maintained stability in alkaline conditions [18]. Many proteases derived from *Trichoderma* or related fungal species have demonstrated the optimal activity within more narrow temperature ranges, often between 25 °C and 35 °C, with significant loss of activity at temperatures exceeding 40 °C [27]. For instance, *T. harzianum* proteases, frequently studied for their biocontrol properties, tend to lose activity at higher temperatures, which can be a limitation in warmer climates or under fluctuating environmental conditions [28]. However, the recombinant PRA1 protease in this study showed considerable resilience and remained functional even at 45 °C, retaining more than 60% of its enzymatic activity. This robustness sets it apart as a more versatile enzyme capable of being effective in different agricultural settings, where temperature fluctuations are common [29]. 

In this study, metal ions such as Fe^2+^, Fe^3+^, Cu^2+^, and Mn^2+^ inhibited the enzyme’s activity, while Ca^2+^ and Mg^2+^ promoted its activity. Na^+^, K^+^, Li^+^, and Zn^2+^ had no significant effects on its activity under the optimal reaction conditions for the recombinant PRA1 protease (35 °C and pH 8.0). Transition metals such as iron and copper are known to participate in redox reactions, and their presence may alter the oxidation state of amino acid residues in the active site, leading to a reduction in catalytic efficiency [30]. Ca^2+^ in particular has been shown to enhance the activity of proteases by stabilizing the conformation of the enzyme [31]. Consistent with previous studies on SprT protease [18], metal ions like Ca^2+^ and Mg^2+^ activated its activity, while Fe^2+^ inhibited it. Additionally, TaproA1 protease, discovered in *T. asperellum*, was shown to be inhibited by Fe^3+^ and Fe^2+^, respectively [32].

The catalytic mechanism of the *T. harzianum* protease PRA1 was determined using standard inhibitors. At a concentration of 1 mM, PMSF strongly inhibited the enzyme activity by 78%, indicating that PRA1 belongs to the serine protease family [15]. Pepstatin A (0.02 mM) completely inhibited the activity of TaproA1 from *T. asperellum*, indicating that it is an aspartic protease [32]. In this study, the inhibitor PMSF had a strong inhibitory effect on the PRA1 protease, with more than 70% of enzyme activity inhibited at a concentration of 1 mM, further confirming its classification as a serine protease. The recombinant PRA1 protease was identified as S1 family trypsin-like serine protease by bioinformatics analysis. In the current study, the recombinant PRA1 protease displayed the highest hydrolytic activity toward casein, followed by skimmed milk. The enzyme also demonstrated the hydrolytic activity on bovine serum albumin (BSA), gelatin, and nematode eggshells. Multiple nematicidal serine proteases have been purified and cloned from parasitic fungi. Examples include pSP-3 from *Paecilomyces lilacinus* [33], VCP1 from *Pochonia chlamydosporia* [34], and Ver112 from *Lecanicillium psalliotae* [35]. These proteases are classified as subtilisin-like serine proteases, exhibiting a wide range of protein substrates such as casein, gelatin, and eggshells. These enzymes are essential for the degradation of nematode cuticles, resulting in nematode mortality [36].

In the current study, the recombinant PRA1 protease effectively degraded the eggs and inhibited normal hatching. On days 8 and 10, significant egg content leakage caused the eggshells to shrink into empty shells, preventing normal hatching. In addition, the egg hatching inhibition rate of 70.60%, on day 10. Supporting our findings, culture filtrates of *T. virens* G1–3 were shown to inhibit *M. incognita* egg hatching [37]. Additionally, the purified serine protease SprT from *T. pseudokoningii* SMF2 showed high nematicidal activity against *M. incognita* J2s. After SprT treatment for 24 h, the mortality of J2s reached 37.3%. In addition, it also had a significant inhibitory effect on the egg hatching. After 15 days of treatment, the hatching rate of eggs was only 13.64% [18]. The nematicidal activity of *Trichoderma* species is often the result of the synergistic action of various enzymes. Study has shown that several proteolytic enzymes present in the culture filtrates of *T. harzianum*, along with chitinolytic enzymes, work together to degrade the chitin–protein layer of nematode eggshells, thereby disrupting the normal embryonic development of *C. elegans* [38]. The nematicidal potential of *Trichoderma* species suggests that they hold great promise for biological control of nematodes. It has been reported that many protease virulence factors from bacteria are also toxic to nematodes. The findings of greenhouse trials demonstrated that *Bacillus subtilis* GEB5 increased guava plants’ development while simultaneously suppressing the population of *M. enterolobii*, with a 72% reduction [39]. A novel serine protease Sep1 from *B. firmus* DS-1 exhibits nematicidal activity and has been shown to degrade various cuticle-associated proteins of *C. elegans*. For instance, *B. nematocida* secretes neutral proteases Bace16 and Bae16, which specifically target nematodes’ intestinal tissues [40].

Despite the promising findings of this study regarding the nematicidal activity of the recombinant PRA1 protease from T6, some limitations must be noted. This study’s presented the laboratory conditions may not accurately reflect real-world environments, where factors like soil composition, microbial interactions, and temperature fluctuations could affect the protein’s efficacy. In addition, this study focused solely on *H. avenae* eggs. Therefore, testing on a broader range of nematodes would be necessary to assess its general applicability as a nematicidal agent.

## 5. Conclusions

This study marks the first successful purification and characterization of recombinant PRA1 protease using an *E. coli* BL21 (DE3) expression system, demonstrating its higher nematicidal potential. PRA1 effectively degraded *H. avenae* eggshells, preventing J2s development by causing eggshell degradation and content leakage. The use of *E. coli* BL21 (DE3) for production offers a scalable and cost-effective method for commercial applications. This study highlights PRA1’s key role in disrupting nematode eggs’ development, crucial for early nematode control and reducing reliance on chemical pesticides. Overall, our findings advance biological nematode control and lay a foundation for broader application in sustainable agriculture.

## Figures and Tables

**Table 1:** Primers used in this study.

Primer Name	Primer Sequence (5′ to 3′)
YF	ACGCGGATCCATCCAGCCCCGTGGCGCCGA
YR	ACCGCTCGAGTGCGAGGTTCTGGTTGATGTAGTTG
T7	TAATACGACTCACTATAGGG
T7ter	GCTAGTTATTGCTCAGCGG

The underlined sections in the primers represent the restriction enzyme sites of *BamH* I and *Xho* I, respectively.

**Table 2:** Effects of metal ions and inhibitors on the activities of recombinant PRA1 protease.

Reagents	Concentration (mM)	Relative Activity (%)
Control	-	100.00 ± 2.18 c
Fe^2+^	5	41.80 ± 1.43 g
Fe^3+^	5	39.70 ± 0.69 g
Cu^2+^	5	62.56 ± 0.66 f
Mn^2+^	5	69.17 ± 1.34 e
Ca^2+^	5	133.83 ± 0.99 a
Mg^2+^	5	123.16 ± 1.45 b
Na^+^	5	100.30 ± 0.91 c
K^+^	5	101.05 ± 1.04 c
Li^+^	5	101.95 ± 0.94 c
Zn^2+^	5	102.71 ± 0.40 c
EDTA	5	96.39 ± 1.73 d
PMSF	1	25.26 ± 0.69 h

The PRA1 protease’s activity in the absence of any additions was regarded as control. The data are presented as the mean ± SE. Columns with distinct letters exhibited significant differences at *p* < 0.05, as determined by Duncan’s multiple range test.

**Table 3:** Effects of substrates on the activities of recombinant PRA1 protease.

Substrate	Concentration (%)	Relative Activity (%)
Casein	1	100.00 ± 1.88 a
Skimmed milk	1	78.94 ± 1.15 b
Bovine serum albumin	1	25.77 ± 1.66 c
Gelatin	1	23.12 ± 1.45 c
Nematode eggshell	1	26.07 ± 1.79 c

The data are presented as the mean ± SE. Columns with distinct letters represent significant differences at *p* < 0.05, as determined by Duncan’s multiple range test.

**Table 4:** Hatching inhibition rate on eggs treated with recombinant PRA1 protease (10 d).

Sample	Inhibition Rate of Eggs Hatching (%)	Relative Inhibition Rate of Eggs Hatching (%)
PRA1 protease	70.60 ± 3.57 a	66.58 ± 4.06
*E. coli* BL21 (DE3) solution of pET-32a empty vector (Positive control)	10.88 ± 0.67 b	-
Sterile water (Negative control)	12.04 ± 1.64 b	-

Data are the mean ± SE. Columns followed by different letters were significantly different at *p* < 0.05 based on Duncan’s multiple range test.

## Data Availability

The original contributions presented in the study are included in the article, further inquiries can be directed to the corresponding author.
